# Clinical Outcome and Mechanisms of Deep Brain Stimulation for Obsessive-Compulsive Disorder

**DOI:** 10.1007/s40473-015-0036-3

**Published:** 2015-03-11

**Authors:** Maarten van Westen, Erik Rietveld, Martijn Figee, Damiaan Denys

**Affiliations:** 1 Department of Psychiatry, Academic Medical Center, Meibergdreef 9, 1105 AZ Amsterdam, The Netherlands; 2 Amsterdam Brain and Cognition Center, University of Amsterdam, Nieuwe Achtergracht 129 (Building L), 1018 WS Amsterdam, The Netherlands; 3 The Netherlands Institute for Neuroscience, Royal Netherlands Academy of Arts and Sciences, Meibergdreef 47, 1105 BA Amsterdam, The Netherlands; 4 Department of Philosophy, Institute for Logic, Language and Computation, University of Amsterdam, Science Park 107, 1098 XG Amsterdam, The Netherlands

**Keywords:** Deep brain stimulation, Obsessive-compulsive disorder, Clinical outcome, Quality of life, Mechanisms of action

## Abstract

Clinical outcome of deep brain stimulation (DBS) for obsessive-compulsive disorder (OCD) shows robust effects in terms of a mean Yale-Brown Obsessive-Compulsive Scale (YBOCS) reduction of 47.7 % and a mean response percentage (minimum 35 % YBOCS reduction) of 58.2 %. It appears that most patients regain a normal quality of life (QoL) after DBS. Reviewing the literature of the last 4 years, we argue that the mechanisms of action of DBS are a combination of excitatory and inhibitory as well as local and distal effects. Evidence from DBS animal models converges with human DBS EEG and imaging findings, in that DBS may be effective for OCD by reduction of hyperconnectivity between frontal and striatal areas. This is achieved through reduction of top-down-directed synchrony and reduction of frontal low-frequency oscillations. DBS appears to counteract striatal dysfunction through an increase in striatal dopamine and through improvement of reward processing. DBS affects anxiety levels through reduction of stress hormones and improvement of fear extinction.

## Introduction

Obsessive-compulsive disorder (OCD) affects 2 % of the general population [1], causing severe functional impairment as a result of anxiety, persistent intrusive thoughts (obsessions), and behavioral inflexibility (compulsions). A small fraction of these patients is unresponsive to standard treatment regimes with cognitive behavioral therapy (CBT), multiple serotonin reuptake inhibitors (SRIs), clomipramine, and addition of antipsychotics [2]. For a subset of these treatment refractory patients, ablative and neuromodulatory strategies are the last options. Recently, deep brain stimulation (DBS) has been approved as a reversible alternative for ablative stereotactic neurosurgery or gamma-knife radiotherapy [3••]. DBS is a chronic and invasive neuromodulatory technique with the unique possibility of fine tuning its effects. DBS involves stereotactic implantation of electrodes in deep brain structures [4], followed by a trial and error process of optimizing parameter settings through telemetry in response to the patient’s feedback and clinical scores.

This article critically reviews recent evidence on DBS for OCD in order to bring the reader up to date with respect to (1) clinical outcome in terms of Yale-Brown Obsessive-Compulsive Scale (YBOCS) and quality of life (QoL) and (2) recent (January 2011–September 2014) clinical and preclinical evidence on mechanisms of action of DBS. We will discuss side effects elsewhere. We conducted an electronic literature search covering journals with indexations in Embase, PsycINFO, and PubMed as well as the Cochrane Library and the WHO trial register, using the text words “obsessive-compulsive disorder” and “deep brain stimulation.” Because this combination yielded a limited amount of hits and using more specific search terms would introduce more selection bias, we decided to manually select relevant studies. All primary clinical outcome studies as well as case reports were included. All clinical and preclinical studies on mechanisms of action from 2011 onward were included. We selectively included articles before 2011 in so far as they helped to sketch the background for our discussion of the recent studies.

## Clinical Outcome

We identified 23 trials [5–27], and case reports that assessed clinical outcome in terms of YBOCS scores of DBS for OCD were included (see Table 1). It concerns 108 uniquely implanted patients, of which 49 were included in the studies of Mallet et al. [22], Denys et al. [19], Goodman et al. [8], and Huff et al. [18] that used a double-blind sham-controlled crossover design. Five targets were used, including white matter tracts in the anterior limb of the internal capsule (ALIC) or ventral capsule (VC) and the inferior thalamic peduncle (ITP) and gray matter structures in the ventral striatum (VS), the nucleus accumbens (NAc), and the subthalamic nucleus (STN) [28]. Follow-up varied between 6 and 36 months.

**Table 1:** Clinical outcome studies on DBS for OCD

	Year	Design	Follow-up (m)	No.	Area	Mean YBOCS baseline	Mean YBOCS last follow-up	Mean YBOCS reduction BL vs. FU (%)	Mean YBOCS on vs. sham (% change vs. BL), *p* value	Responders (>35 %)	Mean GAF change	Comorbidity
Greenberg et al. [5]	2008	Multicenter^a^	24	26	VC/VS	34.0	20.9	38.5		16 (61.5 %)	24.2	MDD (21), PD (2)
Nuttin et al. [6]^a^	2003	2 × 3 months, crossover, no sham	21	4	ALIC	32.3	19.8	39.0		3 (75 %)		MDD (2), somatoform (1)
Greenberg et al. [7]^a^	2006	Open label	36	10	VC/VS	34.6	22.3	36.0		4 (50 %)	17.2	MDD (8)
Goodman et al. [8]^a^	2010	Staggered onset, 30 or 60 days; sham	12	6	VC/VS	33.7	18.0	46.5	Y-FU/Y-BL, *p* = 0.0392 (no group effect, *p* = 0.904)	4 (67 %)		MDD (all)
Nuttin et al. [9]	1999	Open label	21	4	ALIC							
Anderson and Ahmed [10]	2003	Open label	10	1	ALIC	34.0	1.0	97.0		1 (100 %)		
Aouizerate and Cuny [11]	2004	Open label	15	1	VC/VS	30.0	16.0	47.0		1 (100 %)		MDD
Abelson et al. [12]	2005	4 × 3 weeks (2 on, 2 off), no sham	10	4	ALIC	32.8	23.0	29.9	26.5 (19.8)/29.3 (10.5)	2 (50 %)		MDD (3), BDD, tic disorder
Roh et al. [13]	2012	Open label	24	4	VC/VS	37.0	14.8	59.5		4 (100 %)	15	MDD (3)
Tsai et al. [14]	2012	Open label	15	4	VC/VS	36.3	24.3	33.0		1 (25 %)	13.5	MDD (3), bipolar I (1)
Plewnia et al. [15]	2008	Open label	48	1	ALIC re	32.0	25.0	22.0		0 (0 %)	18	Schizophrenia
Burdick et al. [16]	2010	Open label	30	1	ALIC-NA	31.0	31.0	0.0		0 (0 %)		Tourette^b^
Sturm [17]	2003	Open label	24	4	r NAc					Nearly total recovery, 3 vs. 4 (75 %)		
Huff [18]	2010	2 × 3 months, on/sham, crossover	12	10	r NAc	32.2	25.4	21.0	27.9 (13.4)/31.1 (3.4), *p* = 0.2	1 (10 %)	16.5	
Denys et al. [19]	2010	8 months, open > 2 × 2 weeks, on/sham, crossover	21	16	NAc	33.7	16.2	51.0	21.1 (37.4)/30.0 (11.0), *p* = 0.004	9 (56.3 %)		MDD (6), dysthymic (1), panic (1)
Neuner et al. [20]	2009	Open label	36	1	NAc	32.0	14.0	56.3		1 (100 %)		Tourette^b^
Franzini et al. [21]	2010	Open label	24	2	NAc	34.0	21.0	38.0		1 (50 %)	20	Bipolar I (1) MDD (1), BDD (1)
Mallet et al. [22]	2008	Multicenter, 2 × 3 months, on/sham, crossover, washout	10	17	STN	30.0		31.0	19 ± 8 (36.7)/28 ± 7 (6.7), *p* = 0.01	Uses 25 % as cutoff, 50 % response	13	MDD (2)
Chabardes et al. [23]	2013	Open label	6	2	STN			56.0		1 (50 %)		Substance abuse (1), OCPD
Mallet et al. [24]	2002	Open label	6	2	STN	24.5	4.5	81.6		2 (100 %)		PD^b^
Fontaine and Mattei [25]	2004	Open label	6	1	STN	32.0	1.0	97.0		1 (100 %)		Dysthymic, hypomanic episode, PD^b^
Barcia et al. [26]	2014	Open label	1	2	STN + NAc	33.0	9.5	71.2		2 (100 %)		IQ 70 (1)
Jiménez-Ponce et al. [27]	2009	Open label	12	5	ITP	35.0	17.8	51.0		5 (100 %)	50	Substance abuse (3)

All case reports and clinical trials of DBS for OCD (with YBOCS >28) in humans are listed, grouped by target. All patients have been reported once, except for the patients in the studies indicated with superscript letter a, who have also been included in the multicenter study of Greenberg et al. (2010). Mean Yale-Brown Obsessive-Compulsive Scale (YBOCS) reduction is based on mean YBOCS at baseline (Y-BL) and mean YBOCS at last follow-up (Y-FU). Mean YBOCS scores from on and off/sham conditions are given with *p* values to show whether or not a placebo effect was observed

*GAF* Global Assessment of Functioning Scale, *MDD* major depressive disorder, *BDD* body dysmorphic disorder, *PD* Parkinson’s disease, *OCPD* obsessive-compulsive personality disorder

^a^Patients in these studies have been included in the multicenter trial of Greenberg et al. [5]. In the current study they are represented once

^b^Indicates case reports with comorbidity as the primary indication for DBS

Clinical outcome for all studies, together with each uniquely implanted patient weighted once, yields a mean YBOCS reduction of 47.7 % and of 37.4 % for only the four randomized sham-controlled trials (RCTs). The mean response percentage (minimum 35 % YBOCS reduction [2]) is 58.2 % for all studies and 43.8 % for the double-blind sham-controlled studies, leaving out the study of Mallet et al. that used a lower cutoff point of 25 % [22]. Placebo effects appear to be minimal, as three out of four RCTs demonstrate significant difference between sham and stimulation conditions. VC/VS and bilateral NAc targets, although more extensively studied, show greater symptom improvement than the STN, and bilateral NAc stimulation is more effective than right unilateral NAc stimulation.

One presumed meta-analysis [29] miscalculated effects and had considerable inclusion bias [30]. We argue that differences in the designs of available studies currently obstruct the conduction of a meta-analysis. Compatibility might be increased through consistent use and publication of end points and response rates. The same goes for standardization of parameter setting optimization of which the additional effect is difficult to apprehend. One should also note that the high prevalence of comorbid major depressive disorder (MDD) may mediate effects on OCD. Lastly, more RCTs as well as comparative studies between various targets, especially between VC/VS and STN, are necessary.

### Quality of Life

Half of the DBS studies assessed Global Assessment of Functioning (GAF) scores as a measure of QoL, which amounts to a mean DBS-related GAF increase of 21.5 out of 100 points. Interestingly, the study of Huff et al. [18] had a similar mean GAF improvement despite the fact that only one of these patients was a responder in terms of YBOCS, suggesting that DBS may improve QoL independent of symptom improvement. Correspondingly, the first clinical study on long-term QoL effects of DBS for OCD found significant improvement on the 26-item WHO QoL Scale-Brief Version (WHOQOL-BREF), even in nonresponders in terms of YBOCS [31]. At baseline, DBS-eligible OCD patients were impaired in all five domains compared with age- and sex-matched healthy controls. The physical and psychological domains, which were most severely impaired at baseline, improved with 23 and 27 %, respectively, at 8 months post-surgery, and with a total of 39.5 % at 3–5 years post-surgery compared to baseline. Improvement in physical and environmental domains correlated with symptom improvement on the YBOCS and with improvement of depression and anxiety on the Hamilton Rating Scale for Depression (HAM-D) and Hamilton Rating Scale for Anxiety (HAM-A). The environmental domain (financial, housing, mobility) also improved significantly with 16 %. Only the social domain did not significantly improve, and this was especially due to patients with long illness durations. The overall score improved with 90 %, approaching the normal range, indicating that patients might in fact regain a normal standard of QoL. It appears, however, that DBS is a chronic treatment and that improvements on QoL disappear after acute DBS cessation. This also causes a relapse of obsessions and compulsions and a rebound of anxiety and depression which exceeds pre-surgery levels with approximately 40 % [32].

## Mechanisms of Action

The mechanisms of action of DBS that mediate the clinical effects discussed above were initially understood as equivalent to the lesion effects of ablative surgery [6]. DBS was thought to locally inhibit excessive pathological activity. In recent years, it has been acknowledged that facilitation of informational signals is crucial. Tellingly, there has been a shift in focus from local effects towards connectivity on the system level [33]. We review clinical and preclinical evidence published in the last 4 years and show that the mechanisms of action of DBS are a combination of excitatory and inhibitory as well as local and distal effects.

### Behavioral Studies

Using selective SRI (SSRI)-validated animal models [34, 35], pathognomonic features of OCD, including cognitive and behavioral inflexibility, attenuated reward processing, and anxiety levels have been assessed in relation to DBS. High-frequency (presumably inhibitory) DBS of the lateral orbitofrontal cortex (OFC) resulted in behavioral inflexibility during a reversal learning task [36], suggesting that local high-frequency stimulation disrupted OFC control over striatal areas, thereby inducing perseverative behaviors. Furthermore, reduced compulsive lever pressing was found with high but not with low-frequency stimulation of the rat’s equivalent of the globus pallidus region, which may be explained by activation of passing corticofugal fibers of the internal capsule [37]. Besides these effects on compulsive behaviors, DBS of several other subcortical areas affected unconditioned or conditioned anxiety. Caudate nucleus (CN)-DBS reduced both forms of anxiety, VS-DBS reduced only conditioned anxiety, while NAc and bed nucleus of the stria terminalis (BNST)-DBS did not have any effect on anxiety [38]. Adding to this, VC/VS-DBS during extinction training reduced fear expression and increased fear extinction, which was associated with an increase in OFC, medial prefrontal cortex (mPFC), and amygdala of activity-associated plasticity markers, such as pERK, c-Fos, and BDNF [39•, 40•]. These findings confirm the idea that DBS and CBT, of which extinction is a central element, act as two complementary treatments. Indeed, a recent open-label and uncontrolled clinical DBS trial revealed that the mean YBOCS score and the mean response percentage (minimum 35 % YBOCS reduction) improved from 25 to 42 % and from 50 to 72 %, respectively, after augmentation with 24 weeks of CBT [41]. DBS might reduce anxiety through modulation of the stress hormones, shown by an increase in plasma glucose levels in an animal study [42] and YBOCS-correlated normalization of hypercortisolism in humans [43]. In sum, DBS animal models suggest that the stimulation of OFC striatal fibers improves a compulsive behavior, with more ventral stimulation being related to reduced fear conditioning and improved fear extinction as well.

### Neurotransmitter Studies

OCD phenomenology, and in particular aberrant reward processing, has been related to dysfunction of dopaminergic neurotransmission [44]. A SPECT study in humans with NAc-DBS found a reduced dopamine D_2/3_ receptor-binding potential, which was correlated with long-term clinical improvement and increased plasma levels of homovanillic acid, a dopamine metabolite [45]. This indicates DBS-induced increase in striatal dopamine, which might imply that previous findings of increased dopamine transporter (DAT) availability and decreased dopamine D_1_, a D_2/3_ receptor density in the striatum of OCD patients [46, 47], did not so much indicate downregulation but intrinsic compensatory dopamine release, which is enhanced by DBS. These effects of DBS on dopamine levels might be indirect. A robust finding is that serotonine reuptake inhibitors (SRIs) are effective in reducing symptoms [48] and influence striatal dopamine levels. Possibly, the serotonergic deficits that are typically found in OCD patients, including decreased SERT availability in thalamic and brain stem regions and increased cortical postsynaptic 5-HT_2A_ receptor availability [46, 47], mediate striatal dopaminergic dysfunction. Recent microdialysis studies in healthy rats with NAc-DBS show a rapid increase of both dopamine and serotonin release in frontal cortex areas (OFC and mPFC) [49], but not in the striatal area (NAc) where the stimulating electrode was located [50]. From local recordings in acute rat brain slices, we know that high-frequency stimulation (140 Hz) of the NAc significantly suppresses spontaneous local neuronal firing. This suppression could be selectively reversed through the application of GABA_B_ and non-GABA_A_ antagonists [51]. Possibly, DBS activates GABA-ergic inhibitory interneurons.

### Imaging Studies

Structural brain imaging of OCD patients reveals increased gray matter volume of striatal areas (CN and putamen) and decreased volume of frontal cortical areas (anterior cingulate (ACC) and OFC) [52]. Functional imaging indicates hyperactivity in both striatal and frontal areas (CN and OFC) in resting state and during symptom provocation [53]. Taken together, OCD pathophysiology implies hyperconnectivity between frontal and striatal areas. This long-standing hypothesis was confirmed by DBS-induced normalization of excessive coupling between frontal cortex and NAc in a recent fMRI study in OCD patients. This was paralleled by a normalization of decreased NAc activity [54•]. Recent studies on clinical cohorts of OCD patients treated with STN-DBS [55] and NAc-DBS [54•] show a similar YBOCS-correlated decrease of activity in frontal and striatal areas as had been observed with treatment with SSRIs or CBT [56]. Hyperconnectivity of brain areas in OCD pathophysiology likely includes hyperactivity or hypoactivity in the limbic system, thalamus, and parietal cortex, too [57, 58]. Recent PET findings of DBS-eligible OCD patients include increased resting state metabolism in ACC, occipital cortex, and posterior cerebellum. Several months of VC/VS-DBS resulted in decreased metabolism in anterior cingulate and prefrontal and orbitofrontal cortices although uncorrelated to YBOCS improvement [59]. Preclinical findings from NAc-DBS in healthy pigs include activation of prefrontal, insular, cingulate, and parahippocampal regions [60]. Together with the above-discussed evidence from behavioral and neurotransmitter studies, these functional imaging findings converge with the hypothesis that DBS improves OCD by restoring corticostriatal function, possibly in a top-down direction through activation of distant neural assemblies [61, 62].

### Electrophysiological Studies

In line with the aforementioned imaging findings, neuronal populations in the striatum of OCD patients show aberrant discharge patterns. Studies on local recordings in the STN through unconnected DBS electrodes in OCD found increased burst firing in STN neurons in resting state. During symptom provocation, synchrony in the discharge pattern of STN neurons is significantly increased [64]. Patients with lower interburst intervals and higher intraburst frequency had the best YBOCS outcome [65]. Moreover, OCD symptom severity could be predicted by STN discharge patterns. The most severe cases were related to a higher intraburst frequency and more low-frequency oscillations where present [65]. The functional role of this aberrant bursting pattern in OCD, for instance its relation to dopamine levels, remains unclear. To our knowledge, no studies combined in vivo local recordings with active STN-DBS or did local recordings in patients after successful DBS treatment. NAc activity has predictive capacity on a short timescale and has been studied under active stimulation. Intraoperative local recordings in OCD and MDD patients during a gambling task revealed that seconds before their decision is physically manifested, NAc activity already significantly predicts whether subjects will bet high or low. If the result is unexpected, NAc activity successively potentiates when positive and attenuates when negative, which might implicate that the NAc, together with the midbrain dopaminergic system, facilitates reinforcement learning [63]. Although imaging studies point that DBS directly modulates this activity remains unclear, as NAc-DBS in acute rat brain slices had no effect on the discharge pattern or synchrony of local NAc neurons [51], and with NAc-DBS, the bursting pattern of dopaminergic neurons in nigrostriatal and mesolimbic areas in healthy anesthetized rats appeared to be unaltered [66]. One study connected direct local recordings of NAc neurons with surface EEG of the frontal cortex and found top-down-directed synchrony from the frontal cortex to NAc in low-frequency bands during reward anticipation in OCD patients [67]. Moreover, low-frequency oscillations are reduced by NAc-DBS, as was shown by EEG recordings both during resting state [68] and symptom provocation [54•]. These strands of evidence support the hypothesis that the hyperconnectivity of frontal and striatal brain areas, observed with functional imaging, is mediated by low-frequency oscillations, which are typically associated with long distance communication in the brain [69].

## Conclusion

Clinical outcome of deep brain stimulation (DBS) for obsessive-compulsive disorder (OCD) shows robust effects in terms of a mean Yale-Brown Obsessive-Compulsive Scale (YBOCS) reduction of 47.7 % and a mean response percentage (minimum 35 % YBOCS reduction) of 58.2 %. Additionally, it appears that most patients regain a normal quality of life after DBS. The interpretation of these findings is limited by variations in study designs with respect to the use of a blinded control condition, brain targets, end points, comorbidity, and parameter setting optimization.

Considering the current evidence, it is most likely that the mechanisms of action of DBS are a combination of excitatory and inhibitory as well as local and distal effects. Evidence from DBS animal models converges with human DBS EEG and imaging findings, in that DBS may be effective for OCD by reduction of frontostriatal hyperconnectivity, which is likely achieved through reduction of top-down-directed synchrony and reduction of frontal low-frequency oscillations. DBS appears to counteract striatal dysfunction through an increase in striatal dopamine and through improvement of reward processing. DBS affects anxiety levels through reduction of stress hormones and improvement of fear extinction. The latter relates to epigenetic changes in frontal cortical areas. Further research needs to elucidate how this relates to DBS-induced increase in frontal dopamine and serotonin levels. The observed distal effects with NAc-DBS, in terms of a decrease in prefrontal cortex metabolism, are also observed in DBS of the STN, which is also connected to frontal cortical areas. The functional role of aberrant bursting patterns of STN neurons in OCD, for instance its relation to dopamine levels, remains unclear. Future studies could combine in vivo local recordings with active STN-DBS or perform local recordings in patients after successful DBS treatment.
